# The Institutional Worker

**Published:** 1913-01-04

**Authors:** 


THE
### INSTITUTIONAL WORKER
### Being a Special Supplement to "The Hospital"

### BUREAU OF INFORMATION.

### Rules for Correspondents.

1. Every letter, on whatever subject, must be accompanied by **the coupon** to be cut from the back cover (inside page) of The Hospital, current issue, and must contain the name and address of the correspondent with pseudonym for publication if desired.

2. Replies by post cannot be given, save under exceptional circumstances at the Editor's discretion.

3. Letters from Approved Homes in reply to special needs published in the Bureau should state terms and full particulars, and be sent prepaid under cover to the Editor of the Bureau with name written across coupon for identification.

4. Proprietors of Homes which have not yet been entered on the List of Approved Homes, but have spare accommodation likely to suit special needs, are invited to write for an application form for registration. The fee for registration, which includes two announcements of the Home in the Bureau, is 5s.

5. Special needs of every description relating to those who are sick or in difficulties are made known in these columns **free of charge.**

6. All communications to be addressed to The Editor of The Hospital, 28 Southampton Street, Strand, London, W.C., and marked "Bureau of Information."

### Needs and Helpers.

**Home for Elderly Lady.**—The patient is sixty years of age, the daughter of a medical man (deceased), and has aneurism of the heart. She has no means, but her friends can raise 10s. a week. She is able to dress and help herself. Write to the Helena Nursing Home, Brownlow Road, Reading. Also to St. Peter's Home, Mortimer Road, Kilburn, and to the Woodside Home, Whetstone, N. The main difficulty is to procure a vacancy, as establishments for this class of patient are much sought after. Let us know whether you succeed.—Mrs. K., Richmond.

**Home for Retired Nurse.**—Comfortable home sought by nurse able to pay 15s. a week. Useful, active, and pleasant companion. In or near a town in dry situation preferred.—Miss O. T. (142x).

### Miscellaneous.

**Extempore Outdoor Treatment.**—We understand that you desire to arrange open-air quarters for a mild tubercular case, but have no balcony available. You must procure or have built an open-air shelter on a concrete base within a convenient distance of the house. The shelter must be connected with the nurse's room by an electric bell, and at first a night visit should always be paid. Any nervousness experienced will soon disappear. In this climate a revolving structure is by far the most convenient, for when the prevailing wind is south for weeks together this saves much discomfort. The patient's comfort will depend much on the provision of ample light and warm bed-coverings. It is important even when no bad symptoms declare themselves to have the patient under the regular supervision of a doctor, for much may be done by graduated exercises and regulated diet to fortify the constitution against the disease. Map out a regular time-table, as is done in sanatoriums, with a view to keeping mind and body fully occupied, and give facilities for following up tastes and talents. No patient who is bored is in a good position for throwing off disease.—Private Home.

**Valuation of Nursing Home.**—Write to the Scholastic, Clerical, and Medical Association, 22 Craven Street, Strand.—K. P.

**Turner Memorial Home.**—This Home is still in existence, and the address is Aigburth, Liverpool.—Miss V.

*** Replies to other queries are unavoidably held over till next week.—Ed.

### Approved Homes Added to the List.—October to December 1912.

In each case direct personal evidence has been received from medical men in regard to the special qualifications of the principals for conducting a Nursing Home for the class of patients received. Former patients or residents in the locality have also contributed their testimony to the satisfactory nature of the accommodation offered. We can, therefore, confidently recommend these Approved Homes to members of the public seeking accommodation in any of the localities indicated.

**Bedfordshire.**—Burnham House, Toddington, near Dunstable. Principals: Mr. and Mrs. A. V. Drewitt Pinchin. Private Home for four mental or epileptic patients (male). Special experience and skill with difficult youths. Healthy, bracing situation, with good garden.

**Brighton.**—114 Marine Parade, Kemp Town. Principal: Miss Janet Reilly. Medical, surgical, maternity, nerve, or permanent cases. Open situation near sea and downs. Wide and successful experience in nursing and in the general administration of a Nursing Home.

**Bucks.**—Braeside, Horn Hill, Chalfont St. Peter. Matron: Miss Crighton. Epileptic and slight mental cases. Backward and delicate children. Outdoor country life; gardening; suitable education; bracing air; large garden.

**Cheltenham.**—St. Neots, Marle Hill Parade, Cheltenham. Proprietor: Martin E. Martin. Vibro or electric massage. Good accommodation for persons requiring rest and home comforts. Pleasant garden, with vinery. Close to spa and park.

**Cheshire.**—One little girl can be received for education with principal's own child in pleasantly situated house in the country. Skilled nursing and healthy open-air life for delicate child. Address K. W. (No. 52), c/o The Bureau.

**Clacton-on-Sea.**—Melrose Nursing and Convalescent Home, Marine Parade. Principal: Miss Kate Heathcote. Medical, surgical, maternity, and convalescent cases. Special advantages for children. South aspect, with broad verandah facing sea.

**Clapham Common, S.W.**—6 Crescent Grove. Principal: Miss Starey. Home for ladies—medical, surgical, and maternity, but principally for the aged. Established nineteen years. Large grounds and country cottage dépendance at Amersham-on-Hill, Bucks. Very moderate terms. Telephone: 429 Battersea.

**Dorset**.—Newland, Sherborne. Principal : Mrs. Marden. Convalescents and those in need of rest and change. Massage as required. Simple life. Moderate terms, specially reduced for nurses. No acute cases.

**Dover.**—7 Waterloo Crescent. Principals : The Misses Elliott. Medical, surgical, rest-cure, maternity cases. Excellent accommodation for permanent patient in good room overlooking sea, with south aspect.

**Harrow-on-the-Hill**.—78 Butler Road. Principals : Mr. and Mrs. Stanley Gilbert. Electrical treatment, massage, and baths, for one patient only, under trained medical electrician and masseur, with latest apparatus. Domestic comfort studied. One or two non-resident patients can be treated.

**Hemel Hempstead**.—Bracken Lodge, 44 Marlowes. Principals : The Misses Eastty. Medical, surgical, midwifery, and convalescent cases. Scalp massage. Special reduction for necessitous nurses. Bracing climate.

**Isle of Wight**.—Landholm, Totland Bay. Principal : Miss Richardson. Medical, surgical, maternity, rest-cure, and other non-infectious cases. Massage as required. Very comfortable winter home at moderate terms, which can be specially reduced for ailing nurses.

**Leatherhead**.—1 Sackville Villas. Principal : Mrs. Day. Slight mental or convalescent cases. Good house, with garden. Cheerful companionship. Moderate terms.

**Lugano**.—Villa Monteverde, Via Bella Vista, Lugano, Switzerland. Principal : Miss Lorraine Heath. Medical, surgical, obstetric, and convalescent cases. Splendid winter climate. English comfortable life abroad. Diet a speciality. Fully trained and certificated nurses.

**Royal Leamington Spa**.—Warneford House, Radford Road, and 37 Clarendon Square. Principal : Mrs. Ward, M.I.H. Medical, surgical, maternity, rest-cure, and convalescent. Special advantages for elderly people and permanent cases.

**Somerset**.—Principals : Doctor and his wife. One patient received. Neurasthenic case or very slight tubercular, or delicate child. Very healthy locality. Home life. Exceptional experience in tubercular cases.

**South Devon**.—West Brimley House, Teignmouth. Principals : Miss Gladwin and Miss Hewlett. Fully equipped Nursing Home for medical, surgical, maternity, and other cases, not mental. Massage. Facilities for open-air treatment. Mild climate. Special care given to delicate children.

**Southampton**.—2 Rockstone Place. Principal : Sister Sarjantson. Medical and nerve cases. Dowsing Radiant Heat treatment. Electric and Swedish massage. Cottage by the sea as annexe.

**Stirlingshire**.—The Manse, Slamannan, N.B. Proprietress : Mrs. Reid. Slight mental and nerve cases or incipient tuberculosis. Rural district, 500 feet above sea-level. Air very favourable for lung cases. Goat's milk if required.

**Surrey**.—Gothic House, Tadworth. Principal : Miss Haines, certified nurse masseuse. Small, comfortable rest-house, with private sitting-rooms; 600 feet above sea-level, on extensive common near Walton Heath Golf Links and Epsom Downs; within an hour of London. Inclusive terms or rooms.

**Westcliff-on-Sea.**—St. Ursula's, King's Road. Principal : Miss Haslock. Slight mental and nerve cases, depression, melancholia, etc. Organised system of employment for patients. Large airy premises, with sea view. Wide and successful experience.

**Woodford Green.**—Forest Hall, Hale End, near Epping Forest. Principals : Miss Garrard and Miss von Limburg. Delicate children and (in separate wing) a few mentally deficient and paralysed. Large garden. Education and skilled nursing.

**Worthing.**—13 St. George's Road. Principal : Miss Jane Green. Delicate, convalescent, elderly, or chronic patients. Special advantages for children. Large room with balcony overlooking sea. Every home comfort.

### Institution Facts and Figures.

### AN INVITATION TO PRACTICAL WORKERS.

### Questions for January.

1. State the method in use in your institution for making either beef-tea or broth, or both. How much does the liquor work out at per pint respectively? State the price paid for the meat, and what saving, if any, is effected by the use of a stock-pot.

2. What difference could you make in the bill of fare for the staff, irrespective of their numbers, if you were allowed an extra shilling per head per week for their board? The original allowance may be estimated at six, seven, or eight shillings per head. It is desired to demonstrate how much could be obtained in luxuries for the extra shilling.

Answers are invited from workers in every class of institution for the sick, and the value of the information collected will be greatly enhanced if opportunity be afforded for contrasting figures in various parts of the kingdom. The name of the institution need not be mentioned unless desired. The only condition is that the figures must be the result of actual observation and computation, not estimates. Either or both of the questions may be answered by each contributor. The best answers will be published, and for each contribution considered by the Editor to be of sufficient interest for publication payment of Five Shillings will be made.

The following rules must be observed by contributors to the Facts and Figures Column :—

1. Answers must be written on one side of the paper. They must bear the name and address of the sender and be accompanied by coupon to be cut from the back cover (inside page) of the current issue of The Hospital. A pseudonym must be chosen if the name is not to be published.

2. Answers must be addressed to the Editor of The Hospital, 28-29 Southampton Street, Strand, London, W.C.; they must be marked in the left-hand corner "Facts and Figures," and must be received before the end of the month.

### Editor's Notices.

**Contributions:**

Contributions should be written, or preferably typed, on one side of the paper only, and all articles sent in are accepted upon the distinct understanding that they are forwarded to THE HOSPITAL only.

The Editor cannot undertake to return MSS. not used, but when a stamped directed envelope is enclosed the MSS. may be returned if a special request is made.

Accepted articles and paragraphs of news will be paid for after publication at the scale rate.

**Address:**

To prevent delay all contributions and letters on editorial business must be addressed exclusively to the Editor, "The Hospital," 29 Southampton Street, Strand, London, W.C. It is important that this regulation shall be strictly observed.

**Correspondence:**

Correspondence on all subjects is invited. The name and address of correspondents must be given as a guarantee of good faith, but not necessarily for publication.

**Special Articles:**

Special articles are invited, and questions, inquiries, and paragraphs upon all matters relating to the work, administration, and management of general, special, mental, fever, cottage and convalescent hospitals, sanatoria, homes, institutions, societies and organisations for the treatment or care of the sick, injured and dependants of all classes. Every matter affecting the interests and welfare of the staff of all grades working in these institutions will receive special consideration.

**Administrative Medicine, Research and Items of News:**

Special terms will be paid for approved articles on subjects in this wide field by experts who have made some section of it their special study and interest. We wish to give prominence to every movement tending to advance accuracy of diagnosis, efficiency in treatment, and the development of modern methods to eradicate disease and promote the welfare of the suffering.

**A Bureau of Information:**

We invite inquiries and applications for information and help in providing for that numerous class of sufferers who require special care or house-room which they cannot obtain in their own homes or secure for themselves. **We cannot, however, prescribe, or recommend practitioners.**

**Books for Review:**

Publishers are particularly requested to send advance proofs of any new books of importance whenever possible, as the Editor has made arrangements to publish immediate reviews on a new plan.

**Photographs, Plans, Blocks, and Illustrations:**

It is requested that wherever possible MSS. may be accompanied by illustrations in any of the above forms. The name of the sender and of the article to which it belongs should be written on the back of each photograph, drawing, or block for purposes of identification.

**Local Papers:**

Newspapers containing reports or news paragraphs should be marked and addressed to the Sub-Editor.

**The Coupon System:**

A coupon will be found at the bottom of the third inside page of the cover each week. This coupon must be attached to every question or inquiry to which an answer is desired in THE HOSPITAL.

### Facts Worth Remembering.

**To the Institutional Worker:**

This Special Supplement affords exceptional facilities to those seeking appointments in any grade or department of the Institutional, Health, and Sanitary Services. In this section a large number of vacancies in Hospitals, Infirmaries, and Institutions of every description throughout the United Kingdom are announced every week. Should you not find a post suited to your needs we would remind you that a column is reserved to those seeking re-engagement, and an announcement of twenty-one words, costing 1s., in this portion of the Journal will enable you to bring your requirements prominently before the Executive Officials of every class of Institution, as well as those responsible for Poor-Law and Municipal administration.

**To Institution Authorities:**

The officials controlling institutions will also find THE HOSPITAL of the greatest practical utility when a vacancy occurs upon their staffs, as an announcement in its columns secures the widest publicity among those trained to Institutional Work, and through its agency it is possible to obtain the most efficient officers in every section of Institutional Life. In these circumstances particular attention is drawn to the fact that SPECIAL RATES are allowed to those Institutions that agree to send during 1913 all their public notices for insertion in this section of THE HOSPITAL.

### Manager's Notices.

**Letters relating to the publication, sale, and advertising departments of "The Hospital" must be addressed to The Manager, "The Hospital," The Hospital Building, 28 and 29 Southampton Street, London, W.C.**

**Subscriptions** may begin at any time, and are payable in advance. Cheques and Post-Office Orders should be crossed London County and Westminster Bank, Covent Garden Branch, and made payable to the Manager of THE HOSPITAL.

RATES OF SUBSCRIPTION (payable in advance).

UNITED KINGDOM.

| | | |
|---|---|---|
| Three Months (including Postage) | ... ... ... ... | 2s. 0d. |
| Six Months do. | ... ... ... ... | 4s. 0d. |
| Twelve Months do. | ... ... ... ... | 6s. 6d. |

FOREIGN AND COLONIAL.

| | | |
|---|---|---|
| Three Months (including Postage) | ... ... ... ... | 2s. 6d. |
| Six Months do. | ... ... ... ... | 5s. 0d. |
| Twelve Months do. | ... ... ... ... | 8s. 8d. |

**Advertisements:**

To ensure insertion, all advertisements for THE HOSPITAL must reach the Manager not later than Wednesday morning in each week. For scale of charges see pages v and 4.

**Remittances:**

**It is especially requested that remittances be made by Cheque or Postal Order, and in halfpenny stamps only when the amount is under sixpence.**

